# Contemporary Risk of Hip Fracture in Type 1 and Type 2 Diabetes: A National Registry Study From Scotland†

**DOI:** 10.1002/jbmr.2118

**Published:** 2013-10-23

**Authors:** Eleanor J Hothersall, Shona J Livingstone, Helen C Looker, S Faisal Ahmed, Steve Cleland, Graham P Leese, Robert S Lindsay, John McKnight, Donald Pearson, Sam Philip, Sarah H Wild, Helen M Colhoun

**Affiliations:** 1 University of DundeeDundee, UK; 2 University of GlasgowGlasgow, UK; 3 Glasgow Royal InfirmaryGlasgow, UK; 4 University of EdinburghEdinburgh, UK; 5 University of AberdeenAberdeen, UK; 6 NHS GrampianAberdeen, UK; 7 NHS FifeKirkcaldy, UK

**Keywords:** EPIDEMIOLOGY, FRACTURE PREVENTION, OTHER DISEASE AND DISORDERS OF OR RELATED TO BONE

## Abstract

The purpose of this study was to compare contemporary risk of hip fracture in type 1 and type 2 diabetes with the nondiabetic population. Using a national diabetes database, we identified those with type 1 and type 2 diabetes who were aged 20 to 84 years and alive anytime from January 1, 2005 to December 31, 2007. All hospitalized events for hip fracture in 2005 to 2007 for diabetes patients were linked and compared with general population counts. Age- and calendar-year-adjusted incidence rate ratios were calculated by diabetes type and sex. One hundred five hip fractures occurred in 21,033 people (59,585 person-years) with type 1 diabetes; 1421 in 180,841 people (462,120 person-years) with type 2 diabetes; and 11,733 hip fractures over 10,980,599 person-years in the nondiabetic population (3.66 million people). Those with type 1 diabetes had substantially elevated risks of hip fracture compared with the general population incidence risk ratio (IRR) of 3.28 (95% confidence interval [CI] 2.52–4.26) in men and 3.54 (CI 2.75–4.57) in women. The IRR was greater at younger ages, but absolute risk difference was greatest at older ages. In type 2 diabetes, there was no elevation in risk among men (IRR 0.97 [CI 0.92–1.02]) and the increase in risk in women was small (IRR 1.05 [CI 1.01–1.10]). There remains a substantial elevation relative risk of hip fracture in people with type 1 diabetes, but the relative risk is much lower than in earlier studies. In contrast, there is currently little elevation in overall hip fracture risk with type 2 diabetes, but this may mask elevations in risk in particular subgroups of type 2 diabetes patients with different body mass indexes, diabetes duration, or drug exposure. © 2014 The Authors. *Journal of Bone and Mineral Research* published by Wiley Periodicals, Inc. on behalf of the American Society for Bone and Mineral Research.

## Introduction

Hip fractures are an important cause of mortality and morbidity, especially in the elderly. They account for the majority of fracture-related health-care expenditure and mortality in men and women over the age of 50 years within the European Union.(1) Case fatality for hip fracture is high, with relative hazard for all-cause mortality in the first 3 months after fracture of 5.75 (95% confidence interval [CI] 4.94–6.67) in women and 7.95 (CI 6.13–10.30) in men.(2)

Previous studies have shown that type 1 diabetes is associated with an increased risk of hip fracture. Vestergaard combined studies in a meta-analysis and concluded that the risk ratio for hip fracture in type 1 diabetes excluding one outlier was elevated sevenfold.(3) The observed risks vary between individual studies, and many studies are very small. There have been more studies in type 2 diabetes, but the results are less consistent. Large studies have generally shown a smaller increase in relative risk compared with the general population(4,5) than found in type 1 diabetes, whereas smaller studies show no significant elevation in risk.(6–8) A systematic review and meta-analysis in 2007 concluded that the relative risk of hip fracture in type 2 diabetes compared with the nondiabetic population was 1.38.(3) However, the majority of published studies for both type 1 and type 2 diabetes are now several years old, and data collection in some cases goes as far back as 1975 in type 1^9^ and 1970 in type 2 diabetes.(6) Over the past 20 years, there have been many changes in the management of diabetes that might alter the relative risks of fracture associated with diabetes; there have been improvements in glycemic control, earlier detection of type 2 diabetes, reductions in the prevalence of renal failure, and changes in drug treatments that may influence fracture risk, eg, introduction of thiazolidinediones.(10) Many of the above studies report relative risks that are an average of risks pertaining to many years and so reflect historical risks.(4,6,9,11,12) To establish the contemporary risks of hip fracture associated with diabetes, we used a nationwide diabetes register and data from the total nondiabetic population for Scotland. We focused on hip fractures because these are comprehensively captured by hospital admission data, our main source of outcome data. Unlike many previous studies, we were able to examine contemporary risks, and unlike previous studies of type 1 diabetes, we were able to examine risks across all age bands, not just younger persons.

## Materials and Methods

### Ethics statement

Approval was obtained from the Scotland A Research Ethics Committee, Privacy (Caldicott) Guardians for the 14 Scottish Health Boards, and the Scottish Privacy Advisory Committee.

### Data sources

In Scotland, a single nationwide clinical information system, the Scottish Care Information-Diabetes Collaboration (SCI-DC) database, has captured registration of patients with diabetes since 2000. Registration occurs automatically when a Read code for diabetes is assigned in primary or secondary care. Because all but 5 of 1076 general practices nationwide contribute data, it is estimated to capture more than 99% of all those with diagnosed diabetes. From a 2008 extract of SCI-DC, we extracted information on all people with type 1 and type 2 diabetes aged 20 to 84 years who were alive at any time between January 1, 2005, and December 31, 2007, inclusive. We included cases prevalent at January 2005 (*n* = 19,083 type 1 and *n* = 144,480 type 2) and cases incident by December 31, 2007 (*n* = 2625 and *n* = 50,673).

We defined type 1 diabetes on the basis of the type of diabetes assigned in the database with the additional requirement that the prescription history did not contradict this (ie, no evidence of lengthy period of diabetes before insulin and no coprescribing of nonmetformin oral diabetes drugs). Type 2 diabetes was defined as either a recorded diagnosis of type 2 diabetes or a diagnosis of type 1 diabetes that was contradicted by clinical history and prescription data. We identified all hospitalized events for hip fracture for type 1 and type 2 diabetes patients in 2005 to 2007 by linkage of the diabetes register to national hospital admissions data (the Scottish Morbidity Record SMR-01) held by the Information Services Division (ISD) of NHS National Services. Death data provided by the National Records of Scotland were used for right censoring follow-up time in those with diabetes. The SMR-01 data set captures all national public sector hospital admissions from 1981 onward.(13) ISD also provided the counts of events and midyear population denominators for the total general population of Scotland aged >20 years for 2005 to 2007. Hip fracture events were defined as hospital admissions with any of the ICD-10 codes S72.0, S72.1, or S72.2 as the main admission reason.

### Statistical methods

Analysis was carried out using STATA 11.1 (StataCorp, College Station, TX, USA). Data for the total population were available in the form of counts of persons with an event in each calendar year, with the corresponding midyear population estimates as an approximation of the person years, broken down by sex, individual age bands, and quintiles of Scottish Index of Multiple Deprivation (SIMD). To obtain counts of persons with events and denominators for the nondiabetic population, we collapsed age into 5-year age bands and subtracted from the midyear total population all those with any type of diabetes at any point in that year and we subtracted from the total count of persons with events all those with diabetes who had an event at any point in that year.

Individual level data on those with type 1 and type 2 diabetes were grouped similarly to give counts of persons with events in each calendar year and the total person-years observed within each calendar year. Age-adjusted incidence rate ratios (IRRs) were calculated, analyzing men and women separately using negative binomial or Poisson models, whichever gave the better fit as assessed by the Akaike Information Criterion.(14) As data on the general population was provided by SIMD, this covariate was included in all analyses. The IRRs associated with diabetes for a given attained age/sex group therefore represent the average effect of diabetes in that group across the 3 years of the study compared with those without any type of diabetes. IRR calculations were restricted to end December 2007 because partial year data for 2008 were not available for the nondiabetic population. Models adjust for the effect of calendar year as a linear trend and adjust for age using 5-year age bands (except that we used a wider 20- to 29-year age band in type 1 diabetes because events were too sparse otherwise in this age band) and deprivation. Where there was evidence of an interaction between the effect of diabetes and age, we estimated the effect of diabetes separately for 10-year age bands while still adjusting for the main effect of age in 5-year age bands. Absolute risk differences were calculated as the difference in age-adjusted rates within age bands between the groups under comparison.

There were relatively few individuals with type 2 diabetes under age 40 years (4966 observed across the 3-year study period, of whom 505 were aged under 30 years). In this group, there was only one event in men in the time period and none in women. At the other end of the age spectrum, we were not able to make an age-adjusted comparison of diabetes with the nondiabetic population aged 85 years and older because the general population data do not provide finer breakdown for that age band. We, therefore, restricted all analyses to subjects aged 20 to 84 years for type 1 diabetes, and 40 to 84 years for type 2 diabetes.

Because body mass index (BMI) is increased in type 2 diabetes but higher BMI is a protective factor for fracture, we also examined the relative risk of hip fracture compared with the nondiabetic population for each tertile of BMI among the type 2 diabetic population. Note that in this analysis the comparator rates are for the nondiabetic population because we did not have BMI data on the nondiabetic population.

## Results

**Fig. 1:**
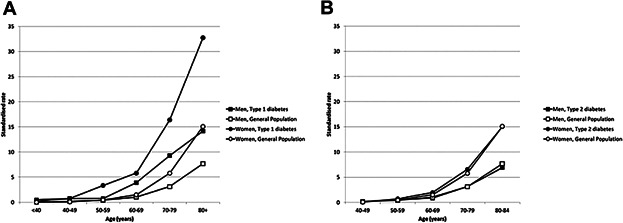
(*A*) Rates of hip fracture in those with type 1 diabetes compared with the nondiabetic population, standardized by age and deprivation category (SIMD). Black square = men with type 1 diabetes; white square = men without diabetes; black circle = women with type 1 diabetes; white square = women without diabetes. (*B*) Rates of hip fracture in those with type 2 diabetes compared with the nondiabetic population, standardized by age and deprivation category (SIMD). Black square = men with type 2 diabetes; white square = men without diabetes; black circle = women with type 2 diabetes; white square = women without diabetes. Note that the age range is 20 to 84 years in type 1 but 40 to 84 years in type 2.

There were 21,033 people with type 1 diabetes aged 20 to 84 years and 180,841 people with type 2 diabetes aged 40 to 84 years observable during the time period described. This equates to 59,585 and 462,120 person-years, and 105 and 1421 events, respectively. This was compared with 3.66 million people and 10,980,599 person-years, with 11,733 events for the general nondiabetic population. Fig. 1 summarizes rates of fracture by age, with the expected marked increase in incidence of hip fracture with age.

### Type 1 diabetes

Table 1 shows the crude rates and IRRs for type 1 diabetes and the nondiabetic population. Type 1 diabetes was clearly associated with an increased risk of fracture in both men and women. The observed IRR for type 1 diabetes in women was similar to the IRR in men (3.54 [95% confidence interval (CI) 2.75–4.57] versus 3.28 [95% CI 2.52–4.26], respectively). Nonetheless, all analyses are stratified by sex because this gives models with better fit and allows for the differing pattern of fracture by age between sexes. The observed IRRs are generally highest in the age bands where the background absolute rates are lower, ie, in the younger populations. The absolute risk difference between men with type 1 diabetes and nondiabetic subjects generally increased with age from 0.42 in those aged 20 to 39 years to 2.78, 5.47, and 7.33 per 1000 person-years in those aged 60 to 69, 70 to 79, and 80 to 84 years, respectively. In women, the absolute risk difference increased from 0.16 in those aged 20 to 39 years to 3.93, 8.07, and 21.88 per 1000 person-years in those aged 60 to 69, 70 to 79, and 80 to 84 years, respectively.

**Table 1:** Incidence Rates and Incidence Rate Ratio (IRR) of Hip Fracture Hospitalization Event in Those With Type 1 Diabetes Compared With the Nondiabetic Population

	Type 1 diabetes			Nondiabetic					
Age band (years)	No. of events	Person-years	Crude rate per 1000 person-years (SE)	No. of events	Person-years	Crude rate per 1000 person-years (SE)	Adjusted^a^ incidence rate ratio	95% confidence interval	*p* Value
Men									
20–39	7	14,391	0.49 (0.18)	143	1,977,521	0.07 (0.01)	6.35	(3.44–11.72)	<0.001
40–49	7	8975	0.78 (0.29)	188	1,101,017	0.17 (0.01)	4.40	(2.48–7.81)	<0.001
50–59	4	5792	0.69 (0.35)	369	930,837	0.40 (0.02)	1.79	(0.60–5.34)	0.299
60–69	11	2877	3.82 (1.15)	697	670,970	1.04 (0.04)	3.79	(2.18–6.56)	<0.001
70–79	11	1280	8.59 (2.59)	1314	420,833	3.12 (0.09)	2.95	(1.88–4.62)	<0.001
80–84	3	200	15.02 (8.67)	872	113,365	7.69 (0.26)	1.96	(0.62–6.18)	0.251
Total (20–84)	43	33,515	1.28 (0.20)	3583	5,214,543	0.69 (0.01)	3.28	(2.52–4.26)	<0.001
Women									
20–39	2	11,407	0.18 (0.12)	39	2,038,897	0.02 (0.00)	8.92	(2.22–35.81)	0.002
40–49	5	6519	0.77 (0.34)	121	1,189,870	0.10 (0.01)	7.47	(3.11–17.93)	<0.001
50–59	13	4104	3.17 (0.88)	459	988,934	0.46 (0.02)	7.13	(4.24–11.99)	<0.001
60–69	13	2397	5.42 (1.50)	1141	767,643	1.49 (0.04)	3.81	(2.69–5.38)	<0.001
70–79	19	1373	13.84 (3.17)	3341	578,686	5.77 (0.10)	2.59	(1.61–4.16)	<0.001
80–84	10	270	36.97 (11.69)	3049	202,026	15.09 (0.27)	2.50	(1.28–4.88)	0.007
Total (20–84)	62	26,070	2.38 (0.30)	8150	5,766,056	1.41 (0.02)	3.54	(2.75–4.57)	<0.001

a Incidence rate ratio is adjusted for age, calendar year, SIMD, and for the overall estimate, an SIMD-age interaction.

### Type 2 diabetes

Table 2 shows hip fracture rates for type 2 diabetes, showing a different pattern in comparison with the nondiabetic population from that observed among people with type 1 diabetes. The overall age-adjusted IRR was 0.97 (95% CI 0.92–1.02), *p* = 0.234, in men and 1.05 (95% CI 1.01–1.10), *p* = 0.013, in women. In men, there was little evidence for an effect of type 2 diabetes in any age group, and the absolute risk differences between type 2 diabetes and nondiabetic populations were small, with the greatest difference (0.84 per 1000 person-years) in the oldest age group. In women, the IRRs for type 2 diabetes varied considerably by age and were statistically different from unity in all but the youngest and oldest of these age groups (Table 2). However, the absolute risk differences were also small, the greatest difference being 0.9 per 1000 person-years for those aged 70 to 79 years. When stratified by tertile of BMI among the diabetic population however, those in the bottom tertile for BMI had higher hip fracture risks than the overall risks in the background nondiabetic population, and those in the upper two tertiles for BMI have significantly lower rates (Table 3). Risks also varied by diabetes duration, with those type 2 diabetes patients in the top tertile for duration (>7 years) having significantly increased risks in both sexes (IRR 1.25 in men [95% CI 1.08–1.45]; IRR 1.55 in women [95% CI] 1.38–1.75).

**Table 2:** Incidence Rates and IRRs of Hip Fracture Hospitalization Event in Those With Type 2 Diabetes Compared With the Nondiabetic Population

	Type 1 diabetes			Nondiabetic					
Age band (years)	No. of events	Person-years	Crude rate per 1000 person-years (SE)	No. of events	Person-years	Crude rate per 1000 person-years (SE)	Adjusted^a^ incidence rate ratio	95% confidence interval	*p* Value
Men									
40–49	4	25,033	0.16 (0.08)	188	1,101,017	0.17 (0.01)	0.87	(0.47–1.61)	0.656
50–59	34	58,347	0.58 (0.10)	369	930,837	0.40 (0.02)	1.14	(0.93–1.39)	0.214
60–69	69	80,582	0.86 (0.10)	697	670,970	1.04 (0.04)	0.87	(0.76–1.00)	0.046
70–79	228	71,606	3.18 (0.21)	1314	420,833	3.12 (0.09)	1.00	(0.94–1.06)	0.921
80–84	119	17,367	6.85 (0.63)	872	113,365	7.69 (0.26)	0.95	(0.86–1.04)	0.248
Total (40–84)	454	252,935	1.79 (0.08)	3440	3,237,022	1.06 (0.02)	0.97	(0.92–1.02)	0.234
Women									
40–49	3	17,258	0.17 (0.10)	121	1,189,870	0.10 (0.01)	1.19	(0.63–2.21)	0.594
50–59	30	37,814	0.79 (0.14)	459	988,934	0.46 (0.02)	1.21	(1.04–1.41)	0.013
60–69	134	61,744	2.17 (0.19)	1141	767,643	1.49 (0.04)	1.14	(1.08–1.21)	<0.001
70–79	468	70,156	6.67 (0.31)	3341	578,686	5.77 (0.10)	1.06	(1.01–1.12)	0.032
80–84	332	22,213	14.95 (0.82)	3049	202,026	15.09 (0.27)	0.99	(0.92–1.07)	0.841
Total (40–84)	967	209,185	4.62 (0.15)	8111	3,727,159	2.18 (0.02)	1.05	(1.01–1.10)	0.013

a Incidence rate ratio adjusted for age, calendar year, SIMD, and for the overall estimate, an SIMD-age interaction.

**Table 3:** Incidence Risk Ratios for Hip Fracture in Type 2 Diabetes Versus Nondiabetic Population by Tertile of BMI in Those With Diabetes

Sex	Tertile	BMI (kg/m^2^)	IRR (95% CI)^a^	*p* Value
Men	1	15.0–27.8	1.34 (1.17–1.55)	<0.001
	2	27.9–32.1	0.63 (0.51–0.76)	<0.001
	3	32.3–79.4	0.48 (0.36–0.62)	<0.001
Women	1	14.1–28.0	1.53 (1.36–1.73)	<0.001
	2	28.1–33.8	0.74 (0.62–0.89)	0.001
	3	33.9–79.9	0.57 (0.45–0.73)	<0.001

a Reference category is the total nondiabetic population.

## Discussion

This study gives a contemporary picture of the risk of hip fracture in people with diabetes. Although our results clearly show an increased risk of hip fracture in type 1 diabetes, among those with type 2 diabetes the overall rates of fracture are consistent with those seen in the general population. However, this overall picture in type 2 diabetes masks a more subtle pattern of risks in subgroups; among those with type 2 diabetes, elevated relative risks are seen in both sexes in those with longer diabetes duration and in those in the bottom tertile for BMI. The strengths of this study lie in the large sample size and comprehensive capture of those with diabetes in Scotland, which enable the risks measured to be truly representative and free of selection bias. The large sample size and recent time span of the data have allowed us to provide accurate up-to-date estimates of risk. However, even with our large sample size, sparse numbers of events in lower age groups mean the results need to be interpreted with some caution for these age bands.

The body of work on type 1 diabetes and fracture risk is small, mostly because of the comparatively low prevalence of type 1 diabetes. Many studies are limited by very small sample sizes and low event rates, such as the Tromsø study,(8) which had only 81 people with type 1 diabetes and only 8 nonvertebral fractures in the 6 years of follow-up from 1994 to 2001 with resulting estimates of risk with wide confidence intervals (17.8 [5.6–56.8] in men, 8.6 [1.2–61.5] in women) compared with those without diabetes. The study covered a wide range of ages from 25 years upward. Similarly, the Nurses' Health Study (1976 to 2002), although large overall, had only 18 women with type 1 diabetes.(12) Here the age-adjusted relative risk (RR) for hip fracture was 7.1 (4.4–11.4), which is higher than our estimate. The population was older than the one presented here, with a mean age over 55 years. Nicodemus and Folsom found 5 fractures in 47 postmenopausal women with type 1 diabetes (a relative risk of 12), in a total population of 32,089 in the Iowa Women's Health Study followed from 1986 to 1997 with an entry age of 55 years upward.(11) These earlier studies have all found higher risks than we find but will not reflect recent changes in the management of type 1 diabetes and are consequently unlikely to represent a contemporary estimate of risk.

The only study to show a lower risk than described here was Vestergaard and colleagues, who in 2000 in Denmark performed a large case-control study including 10,530 hip fractures from the national discharge registry with linkage to national prescription data.(15) Diabetes was ascertained from the discharge registry as well as prescription data. An odds ratio of 1.7 (1.31–2.21) for hip fracture in type 1 diabetes versus no diabetes was reported.(15) It is not clear why these lower relative rates were found in this study; the authors discuss the possibility of misclassification of diabetes in controls. Certainly we know that type 2 diabetes patients on insulin are often mislabeled as having type 1 diabetes; this could reduce the odds ratio in that study but only if it were differential between cases and controls. The only prospective study of a size comparable to ours is Miao and colleagues, who enrolled 24,605 people with type 1 diabetes from 1975 to 1988 in Sweden. The mean age at enrollment was 20.7 years (SD 10.9), and there was a mean 10-year follow-up.(9) Hospitalization ratios (SHR) standardized for age, sex, and calendar year for hip fracture in the population with type 1 diabetes was 7.6 (5.9–9.6) in men and 9.8 (7.3–12.9) for women compared with the nondiabetic population. The risk ratios were higher for those aged over 40 years. We found overall relative risks lower than that reported by Miao and colleagues and that the relative risks fell with age. Vestergaard^3^ combined studies, excluding one outlier study, in a meta-analysis and concluded that the risk ratio for hip fracture in type 1 diabetes was 6.94 (95% CI 3.25–14.78). We have shown in our study that type 1 diabetes is associated with a substantially increased risk of hip fracture, emphasizing the need for a greater understanding of the cause of increased fracture and preventive strategies in type 1 diabetes. However, our data also show that the relative risks are nowhere near as large as in some earlier studies. Because we found that relative risks are lower with older attained ages, some of the differences may be owing to a more truncated age distribution in some of these earlier studies. However, the differences are sufficiently large as to suggest that improvements in type 1 diabetes management may have resulted in a narrowing of the excess risk associated with type 1 diabetes. We cannot assess this directly, but certainly previous studies^8,11^ have suggested that the greatest risk elevation is found in those with more nephropathic and cardiovascular complications, so measures to reduce these might be expected to have impacted on the fracture risk, too. However, whether improved glycemic control, blood pressure management, or increased statin use, all of which have occurred,(16) or some other aspect of management may be responsible is not known.

Studies in type 2 diabetes patients specifically are more frequent and have larger populations. Despite this, they still often derive estimates over a long period of time so that the risk may not necessarily reflect contemporary risks. The risks reported from these studies of people with type 2 diabetes vary substantially. Melton and colleagues, for example, followed their population from 1970 until 1994, showing no significant effect of diabetes on fracture in women over that time period, and a small increase in risk for men.(6) Giangregorio and colleagues^4^ observed 3518 men and women with diabetes and 36,085 controls aged over 50 years with valid FRAX scores from 1990 to 2007, and showed an age-related hazard ratio after adjusting for FRAX score risk factors of 6.27 (3.62–10.87) under 65 years and 2.2 (1.71–2.90) aged 65 years and over. The Cardiovascular Health Study recruited patients aged over 65 years from 1989 to 1993, then rerecruited in 2005 to 2006.(7) Of 5641 people, 918 had diabetes of unspecified type, contributing 8428 person-years of diabetes. Age, sex, and race-adjusted hazard ratio was 1.05 (0.80–1.39). Bazelier and colleagues matched 180,049 people with type 2 diabetes to 490,147 nondiabetic controls, aged over 18 years, with mean of follow-up of 5.3 years for those with diabetes and 6.2 years for controls from 1996 to 2007. They found the same incidence of fractures in both groups (3.1%), but a hazard ratio of 1.21 (1.18–1.23) for those on antidiabetic medication versus nondiabetics after adjustment for age and sex.(5) The Tromsø study found slightly higher but nonsignificant hazard ratios in the population with type 2 diabetes (1.5 [0.5–4.0] in men, 1.7 [1.0–3.0] in women) from 1994 to 2001.(8) Nicodemus and Folsom^11^ showed an age-adjusted relative risk of hip fracture of 1.75 (1.25–2.43) in type 2 diabetes compared with the nondiabetic population. These data were collected from 1986 to 1997 in a population aged over 60 years, so the higher age of the population and the protracted period of data acquisition make comparison with contemporary data difficult. The Nurses' Health Study showed elevated RRs for type 2 diabetes as well as type 1 diabetes, (age-adjusted RR for hip fracture 1.7 [1.4–2.0] in type 2 diabetes) from 1998 to 2002.(12) Again, the data are not contemporary and the population significantly older, so comparisons are limited. The Women's Health Initiative study has a larger population with type 2 diabetes but covered an earlier time period (1975 to 1998), and was also limited to an older population (mean age at screening was over 64 years). They showed a modest increase in age-adjusted relative risk for hip fracture of 1.41 (1.17–1.70).(17) Vestergaard and colleagues in Denmark in 2000 performed a case-control study of fractures including 10,530 hip fractures, and showed an odds ratio of 1.4 (1.2–1.6) for those with type 2 diabetes compared with no diabetes.(15)

Vestergaard also summarized the extensive literature up to 2007 in type 2 diabetes in a meta-analysis and concluded that the relative risk for hip fracture was 1.38 (1.25–1.53).(3) That analysis drew attention to the heterogeneity in relative risks for overall fracture risk in type 2 diabetes. The meta-analysis also reported decreased bone mineral density in type 1 diabetes and increased bone mineral density in type 2 diabetes. Another meta-analysis found a higher summary relative risk for hip fracture of 1.7 (1.3–2.2), possibly reflecting the use of confounder-adjusted estimates.(18) The observed differences in relative risk between the oldest and most recent studies discussed were small, and none of the above studies examined in detail whether there was a time trend in the data available. Thus the estimates from meta-analyses of hip fracture relative risk in type 2 diabetes, mostly encompassing study periods much earlier than our study, show an elevated risk for hip fracture in type 2 diabetes compared with our finding of a lack of elevation in risk in men and a much smaller elevation in women. It is possible that our results reflect an improvement in relative risk associated with type 2 diabetes, but we cannot test this directly. There are several factors that might be expected to operate to alter the risks among people with type 2 diabetes in either direction; for example, type 2 diabetes is getting detected earlier so that our lower risks might reflect a greater prevalence of patients with shorter durations than earlier studies; this would lower relative risks because longer duration of diabetes has been associated with increased risks,(3) and we also found that here. Trends in BMI might also alter the risks through time or between studies. It has been suggested that in type 2 diabetes the elevated BMI may be acting to reduce risk, whereas diabetes per se is increasing risk.(3) Our data are consistent with this idea in that those in the top tertile of BMI had lower risks than the background population and those with the lower tertiles had elevated risks. Relevant to this is that the proportion of our type 2 population who are obese has risen from 44% in 2003 to 55% in 2011.(19) Reduction in complications, especially renal disease risk, might also impact. However, there are factors operating that would be expected to increase risks for fracture in type 2 diabetes, too. In particular, we and others have shown that use of thiazolidinediones is associated with increased hip fracture risk in our type 2 diabetes population.(10)

An important consideration is whether our results are affected by misclassification of exposure or outcome. Details of the sensitivity and specificity of our diabetes registry are provided elsewhere, but in brief we estimate that more than 99% of all diagnosed patients are captured because all but five primary-care practices nationally contribute to the register. The register is used to invite patients for retinopathy screening and less that 4% of invitations are rejected as being sent to a nondiabetic individual, ie, specificity is high. Type of diabetes is validated against diagnosis date and drug history. Of course, undiagnosed type 2 diabetes is not captured, but inclusion of such persons would be expected to result in an even slightly lower relative risk because their duration would be on average less. With respect to hip fracture capture, the data here pertain to hospitalization for hip fracture, so do not capture those who do not present to hospital. However, nonhospitalized hip fracture is rare: In our data, for example, comparing admissions against national death records, we did not identify anyone with hip fracture listed as cause of death without a preceding hospital admission. With regard to coding of hip fracture hospitalizations, a comparison against the Scottish Hip Fracture Audit showed complete capture of hip fractures by hospital discharge codes.(20) Thus we are confident that the data we report are a good representation of risks in people with diagnosed diabetes.

In summary, we conclude that there remains a substantial elevation relative risk of hip fracture in people with type 1 diabetes, but the relative risk is much lower than in earlier studies. In contrast, overall there is currently little elevation in hip fracture risk with type 2 diabetes, but this may mask elevations in risk, in particular subgroups of type 2 diabetes patients with different BMI or diabetes duration or drug exposure. Future work will address key determinants of hip fracture in those with type 1 and type 2 diabetes, allowing further development of individual-level hip fracture risk prediction for those with diabetes.

## Disclosures

HMC has served as a consultant for and received honorarium from Pfizer Inc.; has served on the advisory panel of Sanofi Aventis, Pfizer Inc., Novartis Pharmaceuticals, and Eli Lilly & Company; has received research support from Roche Pharmaceuticals, Pfizer Inc., Eli Lilly & Company, Boehringer Ingelheim, and AstraZeneca LP; has lectured for the speaker's bureau of Pfizer Inc.; and has participated in the following sponsored clinical trials: Researching Cardiovascular Events with a Weekly Incretin in Diabetes (REWIND) study by Eli Lilly & Company, and Reducing with Metformin Vascular Adverse Lesions in Type 1 Diabetes (REMOVAL) study by the Juvenile Diabetes Research Foundation. HMC has also served as a Data and Safety Monitoring Board member for the Novartis trial of ACZ885 Clinical Trial Programme, the Randomized Evaluation of the Effects of Anacetrapib through Lipid-modification (HPS3/TIMI55–REVEAL) trial, and the CANTOS trial of Interleukin-1β inhibitor, as well as on the steering committee for Sanofi Aventis Phase 3 programme for REGN727, a new monoclonal antibody to PCSK9 inhibitor. All other authors state that they have no conflicts of interest.
